# Risk of First Venous Thrombosis by Comparing Different Thrombin Generation Assay Conditions: Results from the MEGA Case–control Study

**DOI:** 10.1055/a-2534-6123

**Published:** 2025-03-19

**Authors:** Kristien Winckers, Eugenia Biguzzi, Stella Thomassen, Alexandra Heinzmann, Frits R. Rosendaal, Tilman M. Hackeng, Astrid van Hylckama-Vlieg

**Affiliations:** 1 Department of Biochemistry, Cardiovascular Research Institute Maastricht (CARIM), University Maastricht, Maastricht, The Netherlands; 2 Department of Clinical Epidemiology, Leiden University Medical Center, Leiden, The Netherlands

**Keywords:** coagulation tests, odds ratio, risk factors, thrombin, venous thrombosis

## Abstract

**Background:**

Hypercoagulability is a risk factor for venous thromboembolism (VTE). Thrombin generation (TG) is a global coagulation assay that measures an individual's clotting tendency. We hypothesise that slow-onset TG (achieved by using a low procoagulant stimulus or an inhibitor of coagulation) is the optimal responsive TG method for detecting hypercoagulability.

This study aimed to compare different TG assay conditions with respect to VTE risk and assess the risk of the first VTE.

**Materials and Methods:**

Basal TG at low tissue factor (TF) concentration and high TF concentration in the presence and absence of activated protein C (APC) were measured in plasma samples from 2,081 patients with first VTE and 2,908 healthy controls from the Multiple Environmental and Genetic Assessment of risk factors for venous thrombosis (MEGA) study. TG parameters and normalised activated protein C sensitivity ratio (nAPCsr) were categorised into quartiles as measured in the controls. We calculated odds ratios (ORs) with 95% confidence intervals (CIs) of the first VTE for different TG categories.

**Results:**

Under all assay conditions the thrombin peak height was associated with VTE risk: peak height of >75th percentile, at low TF OR 6.8 (95% CI 5.5–8.3), at high TF, OR 3.0 (95% CI 2.5–3.6), and at high TF + APC, OR 3.8 (95% CI 3.2–4.5), all compared with a peak height of <25th percentile obtained in controls. An increased nAPCsr (higher resistance to APC) was also associated with VTE risk, OR 3.4 (95% CI 2.8–4.1).

**Conclusion:**

Increased TG is associated with the risk of first VTE, particularly when triggered with a low procoagulant stimulus.

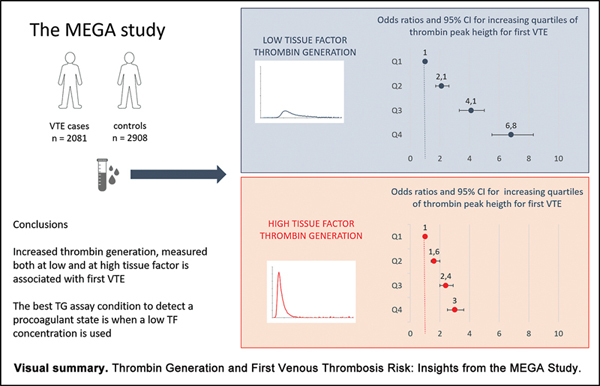

## Introduction

Hypercoagulability or thrombophilia describes an increased tendency of blood to coagulate. Together with endothelial injury and haemodynamic changes, hypercoagulability lies at the base of Virchow's triad, describing the three main pathophysiological factors contributing to thrombosis. To date, there is no golden standard to diagnose hypercoagulability. In clinical practice, physicians often use a plethora of specific assays to detect the most common causes of hereditary and acquired thrombophilia. Commonly used tests include analysis of the factor V Leiden (FVL) mutation, prothrombin mutation, protein S antigen/activity, protein C antigen/activity, antithrombin antigen/activity, lupus anticoagulants, and antiphospholipid antibodies. However, by using this strategy not all forms of thrombophilia are identified. Elevated levels of procoagulant factors such as FVIII, IX and XI are also associated with an increased tendency to form blood clots and will be missed by these specific tests. 1 2 3 Another more general approach to establish hypercoagulability is to search for evidence of in vivo thrombin or fibrin formation. D-dimer levels, thrombin–antithrombin complexes or prothrombin fragments 1 + 2 are markers of thrombin or fibrin formation and indicate recent in vivo clotting activity. Indeed, increased levels are associated with an increased risk of (recurrent) venous thromboembolism (VTE) as is a shorted activated partial thromboplastin time. 4 5 6

Thrombin generation (TG) is a global coagulation test that measures the potential of plasma to generate thrombin. Briefly, the formation of thrombin in a plasma sample is continuously measured by a fluorogenic substrate after initiation of coagulation, typically by tissue factor (TF) in the presence of calcium and phospholipids. 7 The assay has shown to be able to detect thrombophilia both due to inherited and acquired causes. 8 9 10 In TG, endless assay condition variations can be made, affecting the performance of the test to detect hypercoagulable states. We hypothesise that the diagnostic accuracy of the TG assay to detect hypercoagulability can be increased when there is a slow onset of TG. This is particularly the case when a low dose of TF is used or when TG is slowed down by for example activated protein C (APC). Under these circumstances, the tissue factor pathway inhibitor (TFPI)/protein S anticoagulant system is a major determinant of TG, and the designated assay known as the normalised APC sensitivity ratio (nAPCsr) was clinically validated through a strong association with multiple hereditary and acquired risk factors for venous thrombosis. 11 12

In this study, we examined the association between hypercoagulability determined by TG and the first VTE. The first aim of the study was to assess which TG condition is the most responsive to hypercoagulability. For this purpose, TG was measured using three different assay conditions; low TF, high TF, and high TF in the presence of APC (nAPCsr). The second aim of the study was to assess the association between the first VTE and elevated TG.

## Materials and Methods

### Study Design, Patients, and Outcome Measures

The Multiple Environmental and Genetic Assessment of risk factors for venous thrombosis (MEGA) study has been described previously. 13 MEGA is a large case–control study including 4,956 consecutive patients aged between 18 and 70 years with the first episode of VTE (deep venous thrombosis or pulmonary embolism) diagnosed between 1 March 1999 and 31 August 2004. Controls were either partners of patients ( *n* = 3,297) or recruited by random digit dialling ( *n* = 3,000). All study participants filled in an extensive questionnaire on risk factors for VTE at the time of the index date. The index date was defined as the date of diagnosis of the first VTE for the patients. For controls, this was the date of VTE of the partner(s) or the date of filling in the questionnaire (random digit dialing controls). Unprovoked VTE was defined in the absence of trauma, surgery, immobilisation, plaster cast, or pregnancy 3 months preceding the index date, long-distance travel in 8 weeks preceding the index date or the use of oestrogens at the time of the index date. The Medical Ethics Committee of the Leiden University Medical Center approved the study and all participants gave written informed consent.

### Blood Samples

All participants were invited to the anticoagulant clinic to donate a blood sample. For the patients, blood samples were taken at least 3 months after discontinuation of oral anticoagulant drugs or during anticoagulant treatment if treatment was continued 1 year after the event. Blood samples were collected into tubes containing trisodium citrate 0.106 mmol/L and centrifuged at 2,000 × g for 10 minutes after which plasma was frozen and stored in aliquots at −80 °C. For logistic reasons, blood samples were collected until June 2002.

### Thrombin Generation Assays

TG was measured by calibrated automated thrombography (CT) 7 under three different conditions in reaction mixtures (125 μL) containing 80 μL plasma. TG was measured at low TF concentration and high TF concentration (10 pM) in the absence and presence of 5 nM APC. The low TF concentration was chosen in such a way that the thrombin peak height in normal pooled plasma was approximately 40 nM. 14 For the present study, this was achieved at TF concentrations of approximately 2 pm (Dade Innovin, Stago). Coagulation was triggered in the presence of 16 mM CaCl _2_ and 30 μM phospholipids (DOPS/DOPC/DOPE 20/60/20) and measured by the addition of 0.3 mM fluorogenic substrate, all final concentrations. TG parameters such as endogenous thrombin potential (ETP) and peak height were calculated using the Thrombinoscope software 3.0.0.25 (Thrombinoscope, Maastricht, The Netherlands). The nAPCsr was calculated as previously described. 9 The APC concentration was chosen in such a way that the ETP was inhibited for 90% in the normal pooled plasma. To prevent contact activation, contact activation inhibitor thermostable inhibitor of contact activation (TICA) was added to all plasma samples immediately upon defrosting to a final concentration of 30 μg/mL. 15 All thrombin generation parameters were corrected for day-to-day variation using normal pooled plasma, measured in triple on each plate, as a reference as described previously. 9 Normal pooled plasma was made in-house by pooling plasma samples of 23 healthy individuals (57% male subjects, mean age 35 years). The plasma was made by a double centrifugation step (10 minutes at room temperature [RT] at 2,000 × g, subsequently 10 minutes at 11,000 × g) and stored in aliquots at −80 °C before analysis. The intra-assay coefficients of variation were 3.4% and 4.9% for ETP and 3.6% and 5.2% for peak height measured at high and low TF, respectively. The interassay coefficients of variation were 5.3% and 13.3% for ETP and 4.8% and 15.9% for peak height measured at high and low TF, respectively.

### Statistical Analysis

To estimate the effect of baseline characteristics (such as sex, use of oestrogens, age, obesity, FVL mutation, and inflammation) on TG parameters, mean differences in TG parameters were calculated using linear regression analysis in controls only.

Peak height and ETP were measured at low TF and high TF (±APC) and the nAPCsr were categorised into quartiles according to the levels measured in the healthy controls from the MEGA study (<25th [reference category], 25–50th, 50–75th, and >75th percentile). Additionally, the highest quartile of these parameters was further dichotomised at the 90th percentile. To determine the association between TG and first VTE, odds ratios (ORs) with 95% confidence intervals (CIs) were calculated for the different categories of peak height and ETP at low and high TF (±APC) and nAPCSr as estimates of the relative risk. All ORs were adjusted for age and sex. ORs were calculated separately for DVT and PE (with or without DVT). In addition, all analyses were stratified for different strata (non-carriership of the FVL mutation, sex, and hormone use). Statistical analysis was performed using SPSS software.

## Results

Blood samples for TG measurement were available in 2,366 cases and 2,938 controls. Cases ( *n* = 285) and controls ( *n* = 30) using anticoagulant drugs during blood sampling were excluded. Table 1 shows the clinical characteristics of cases and controls. A total of 924/2,081 (44.4%) cases and 1,372/2,908 (47.2%) controls were men. The mean age at inclusion was 48 years for both cases and controls. Venous thrombosis was unprovoked in 687 (33%) cases and provoked in 1,319 (63%) cases. Provoking factors were malignancy ( *n* = 110), surgery ( *n* = 351), plaster cast ( *n* = 103), immobilisation ( *n* = 267), hospitalisation ( *n* = 357), trauma ( *n* = 257) and long-distance travel ( *n* = 354). Seventy-five (4%) cases could not be categorised as provoked or unprovoked as data were lacking. In women, the majority of venous thrombosis (58.6%) occurred while using oral contraceptives. Table 1 shows the TG parameters measured in patients and controls. Under all assay conditions, TG (both ETP and peak height) was increased in cases compared with controls. The nAPCsr was increased in cases as well, indicating a hypercoagulable state due to increased resistance to APC. The lag time of TG was more or less similar in cases and controls. Mean raw thrombin generation parameters obtained in normal pool plasma (at low TF and high TF) were lag time (6.4 and 1.8 minutes), peak height (33 and 211 nM), and ETP (267 and 622 nM·min). The APCsr in normal pool plasma was 1.5.

**Table 1:** Clinical characteristics and thrombin generation parameters in cases and controls

TG parameters	Cases *n* = 2,081	Control subjects *n* = 2,908
Age, years; mean ± SD	47.7 ± 12.8	48.3 ± 12.4
Male/female, *n* (%)	924 (44%)/1,155 (55%)	1,372 (47%)/1,536 (53%)
Risk factors		
OC use	619 (29.7%)	325 (11.2%)
HRT use	58 (2.8%)	88 (3.0%)
Factor V Leiden mutation		
Non-carriers	1,759 (84.5%)	2,765 (95.1%)
Heterozygous carriers	310 (14.9%	137 (4.7%)
Homozygous carriers	11 (0.5%)	6 (0.2%)
Thrombin generation parameters		
Low TF		
Lag time (minutes)	6.83 (2.83)	7.05 (2.47)
Peak height (nM)	44.1 (23.6)	34.8 (23.5)
ETP (nM·min)	409.4 (133.7)	348.1 (148.8)
High TF in absence of activated protein C		
Lag time (minutes)	1.95 (0.40)	1.92 (0.34)
Peak height (nM)	211.9 (39.6)	197.7 (40.0)
ETP (nM·min)	667.6 (130.0)	632.4 (118.0)
High TF in presence of activated protein C		
Lag time (minutes)	3.38 (1.97)	3.33 (0.71)
Peak height (nM)	53.21 (45.58)	33.16 (30.81)
ETP (nM·min)	178.80 (145.37)	119.63 (98.79)
nAPCsr	2.37 (1.89)	1.64 (1.34)

Abbreviations: ETP, endogenous thrombin potential; HRT, hormone replacement therapy; nAPCsr, normalised activated protein C sensitivity ratio; OC, oral contraceptive; SD, standard deviation; TF, tissue factor; TG, thrombin generation.

Table 2 shows the influence of several clinical characteristics on the thrombin peak height and the nAPCSr in healthy controls from the MEGA study. TG and nAPCsr were increased in women compared with men. In women, the use of oral contraceptives or hormone replacement therapy (at the time of blood sampling) was associated with an even more increased TG and nAPCsr ( Table 2 ). Evidently, the presence of the FVL mutation was strongly associated with an increased nAPCsr. However, the mutation had no effect on TG measured in the absence of APC ( Table 2 ).

**Table 2:** Influence of clinical characteristics on thrombin peak height (low tissue factor) and normalised activated protein C sensitivity ratio in control samples

	Thrombin peak height (nM) Mean ± SD		Mean difference (95% CI)	Normalised APC sensitivity ratio Mean ± SD		Mean difference (95% CI)
Sex a	Male *n* = 1,372	Female *n* = 1,238		Male *n* = 1,372	Female *n* = 1,237	
	27.2 ± 12	33.3 ± 18	6.1 (4.9–7.3)	1.40 ± 1.1	2.11 ± 1.4	0.71 (0.61–0.81)
FH user	No *n* = 1,238	Yes *n* = 294		No *n* = 1,237	Yes *n* = 294	
	33.3 ± 18	76.1 ± 37	42.7 (39.8–45.7)	2.11 ± 1.40	3.45 ± 1.5	1.34 (1.16–1.52)
Age, years a	<50 *n* = 1,210	>50 *n* = 1,400		<50 *n* = 1,209	>50 *n* = 1,400	
	32.0 ± 18	28.6 ± 13	−3.4 (−4.6 to −2.2)	1.95 ± 1.5	1.55 ± 1.1	−0.41 (−0.51 to −0.31)
FVL mutation a	Wildtype (FVL ^−/−^ ) *n* = 2,483	Heterozygous carrier (FVL ^+/−^ ) *n* = 121		Wildtype (FVL ^−/−^ ) *n* = 2,482	Heterozygous carrier (FVL ^+/−^ ) *n* = 121	
	30.0 ± 16	32.4 ± 18	2.4 (−0.4 to 5.3)	1.55 ± 1.0	5.20 ± 1.9	3.65 (3.46–3.84)
BMI >30 kg/m ^2^ , ^*^ b	No *n* = 2,194	Yes *n* = 341		No *n* = 2,193	Yes *n* = 341	
	30.1 ± 16	29.7 ± 14	−0.4 (−2.1 to 1.4)	1.74 ± 1.3	1.60 ± 1.1	−0.14 (−0.29 to 0.00)
hsCRP, mg/mL, c	<15 *n* = 2,559	>15 *n* = 46		CRP <15 mg/ml *n* = 2,558	CRP >15 mg/ml *n* = 46	
	30.0 ± 15	38.8 ± 29	8.8 (4.2–13.4)	1.74 ± 1.3	1.59 ± 1.3	−0.15 (−0.53 to 0.24)

Abbreviations: 95% CI, 95% confidence interval; APC, activated protein C; BMI, body mass index; FH, female hormones including oral contraceptives and hormone replacement therapy; FVL, factor V Leiden; hsCRP, high sensitivity C-reactive protein; SD, standard deviation.

a Control subjects using oral contraceptives at the time of blood sampling were excluded.

b Data missing for 31 controls.

c Data missing for three controls.

### Comparison of Different Thrombin Generation Assay Conditions

We measured TG using three different assay conditions. Table 3 shows the ORs for the first VTE for both peak height and ETP measured at low TF and high TF and the latter in the absence and presence of APC. Under all assay conditions, there was a gradual increase in VTE risk for increasing quartiles of both peak height and ETP. The correlation was strongest for peak heights compared with ETP. At low TF concentrations, there was a strong association between TG (both peak height and ETP) and first VTE, whereas the association became weaker at higher TF concentrations. The strong association between TG and VTE measured by the nAPCsr assay, was only slightly decreased in the absence of APC, and this only for the highest quartile ( Table 3 ).

**Table 3:** Distribution of thrombin generation parameters measured at low and high tissue factor concentrations and corresponding odds ratios for first venous thromboembolism

Low TF					High TF − APC				High TF + APC			
Thrombin peak height (nM)												
	Controls *n* (%)	Cases *n* (%)	Crude OR (95% CI)	Adjusted OR (95% CI)	Controls *n* (%)	Cases *n* (%)	Crude OR (95% CI)	Adjusted OR (95% CI)	Controls *n* (%)	Cases ( *n* )	Crude OR (95% CI)	Adjusted OR (95% CI)
Q1	727 (25)	157 (8)	Ref	Ref	727 (25)	260 (13)	Ref	Ref	727 (25)	293 (14)	Ref	Ref
Q2	727 (25)	322 (15)	2.1 (1.7–2.5)	2.1 (1.7–2.6)	727 (25)	423 (20)	1.6 (1.4–2.0)	1.6 (1.4–2.0)	727 (25)	353 (17)	1.2 (1.0–1.5)	1.3 (1.0–1.5)
Q3	727 (25)	624 (30)	4.0 (3.2–4.9)	4.1 (3.3–5.0)	727 (25)	624 (30)	2.4 (2.0–2.9)	2.4 (2.0–2.9)	727 (25)	472 (23)	1.6 (1.3–1.9)	1.8 (1.5–2.1)
Q4	727 (25)	976 (47)	6.2 (5.1–7.6)	6.8 (5.5–8.3)	727 (25)	772 (37)	3.0 (2.5–3.5)	3.0 (2.5–3.6)	727 (25)	961 (46)	3.3 (2.8–3.9)	3.8 (3.2–4.5)
ETP (nM·min)												
	Controls *n* (%)	Cases *n* (%)	Crude OR (95% CI)	Adjusted OR (95% CI)	Controls *n* (%)	Cases n (%)	Crude OR (95% CI)	Adjusted OR (95% CI)	Controls *n* (%)	Cases *n* (%)	Crude OR (95% CI)	Adjusted OR (95% CI)
Q1	727 (25)	180 (9)	Ref	Ref	727 (25)	333 (16)	Ref	Ref	727 (25)	287 (14)	Ref	Ref
Q2	727 (25)	352 (17)	2.0 (1.6–2.4)	2.0 (1.6–2.4)	727 (25)	433 (21)	1.3 (1.1– 1.6)	1.3 (1.1–1.6)	727 (25)	372 (18)	1.3 (1.1–1.6)	1.4 (1.1–1.6)
Q3	727 (25)	627 (30)	3.5 (2.9–4.2)	3.7 (3.0–4.5)	727 (25)	594 (29)	1.8 (1.5–2.1)	1.8 (1.5–2.1)	727 (25)	478 (23)	1.7 (1.3–2.0)	1.8 (1.5–2.2)
Q4	727 (25)	920 (44)	5.1 (4.2–6.2)	5.7 (4.7–7.0)	727 (25)	719 (35)	2.2 (1.8–2.5)	2.1 (1.8–2.5)	727 (25)	942 (45)	3.3 (2.8–3.9)	3.8 (3.1– 4.5)

Abbreviations: 95% CI, 95% confidence interval; APC, activated protein C; ETP, endogenous thrombin potential; N, number; OR, odds ratio; Ref, reference category; TF, tissue factor.

ORs are adjusted for age and sex.

### Thrombin Generation and the Risk of First Venous Thromboembolism

As the peak height at low TF showed the greatest association, we have chosen to further elaborate on this TG parameter in patients with the first VTE. We have compared the test with the nAPCsr, for which the relationship with VTE is well-known. 16 17 For both peak height and nAPCsr, there was a dose-dependent relationship with VTE risk ( Table 3 ). So, at increasing levels of exposure to an increased thrombin generation potency, the risk of VTE increases. A peak height in the highest quartile was associated with a 6.2-fold increased risk (crude OR 6.2; 95% CI 5.1–7.6) for VTE compared with a peak height in the lowest quartile. An nAPCsr in the highest quartile was associated with a 3.0-fold increased risk of first VTE compared with the lowest quartile (crude OR 3.0; 95% CI 2.5–3.5; Table 3 ). For the nAPCsr, the VTE risk was even more increased in subjects with an nAPCsr above the 90th percentile (OR 4.1; 95% CI 3.4–5.0) compared with the lowest quartile. This was not the case for the peak height, as no further increase in the risk of VTE was observed in subjects with peak height above the 90th percentile (OR 5.4; 95% CI 4.3–6.9) compared with a peak height above the 75th percentile both compared with a peak height below the 25th percentile. The adjustment for age and sex did not affect the risk estimates ( Table 3 ).

The data were analysed for provoked VTE, unprovoked VTE, non-carriers of the FVL mutation, men, women, and women not using female hormones at the time of blood sampling separately. The dose-dependent relationship between peak height and nAPCSr and first VTE remained clearly present across these different strata ( Table 4 ). The associations were much stronger for men compared with women. Interestingly, the risks of the first VTE associated with peak height and the nAPCsr were similar for provoked and unprovoked VTE ( Table 4 ). Exclusion of FVL carriers reduced the risk of first VTE in patients with an increased nAPCsr, whereas it did not affect the risk estimates for peak height measured at low TF. This indicates that the increased risk of VTE associated with an increased nAPCsr is partly explained by the presence of FVL carriers, whereas this is not the case for increased TG in general.

**Table 4:** Stratified analysis of the distribution of thrombin peak height and normalised activated protein C sensitivity ratio and corresponding odds ratios for first venous thromboembolism

	Thrombin peak height low tissue factor				Normalised APC sensitivity ratio			
	Controls *n* (%)	Cases *n* (%)	Crude OR a (95% CI)	Adjusted OR b (95% CI)	Controls *n* (%)	Cases *n* (%)	Crude OR a (95% CI)	Adjusted OR b (95% CI)
All subjects								
Q1	727	157	Ref	Ref	726	299	Ref	Ref
Q2	727	322	2.1 (1.7–2.5)	2.1 (1.7–2.6)	727	401	1.3 (1.1–1.6)	1.4 (1.2–1.7)
Q3	727	624	4.0 (3.2–4.9)	4.1 (3.3–5.0)	727	485	1.6 (1.4–1.9)	1.8 (1.5–2.1)
Q4	727	976	6.2 (5.1–7.6)	6.8 (5.5–8.3)	727	892	2.2 (1.9–2.7)	3.4 (2.8–4.1)
Provoked VTE								
Q1	727	85	Ref	Ref	726	133	Ref	Ref
Q2	727	199	2.3 (1.8–3.1)	2.3 (1.7–3.0)	727	229	1.7 (1.4–2.2)	1.6 (1.2–2.0)
Q3	727	358	4.2 (3.2–5.5)	3.9 (3.0–5.0)	727	326	2.4 (2.0–3.1)	2.0 (1.6–2.6)
Q4	727	677	8.0 (6.2–10.2)	6.5 (5.0–8.4)	727	629	4.7 (3.8–5.8)	3.6 (2.8–4.5)
Unprovoked VTE								
Q1	727	68	Ref	Ref	727	151	Ref	Ref
Q2	727	112	1.6 (1.2–2.3)	1.7 (1.2–2.3)	727	150	1.0 (0.8–1.3)	1.3 (1.0–1.6)
Q3	727	244	3.6 (2.7–4.8)	4.1 (3.0–5.5)	727	147	1.0 (0.8–1.2)	1.7 (1.3–2.2)
Q4	727	261	3.8 (2.9–5.1)	6.3 (4.6–8.5)	727	237	1.6 (1.2–2.0)	3.7 (2.9–4.8)
Factor V Leiden, ^−/−^								
Q1	701	134	Ref	Ref	725	298	Ref	Ref
Q2	690	281	2.1 (1.7–2.7)	2.2 (1.7–2.7)	727	400	1.3 (1.1–1.6)	1.4 (1.1–1.7)
Q3	686	522	4.0 (3.2–4.9)	4.1 (3.3–5.1)	720	481	1.6 (1.4–1.9)	1.7 (1.4–2.1)
Q4	688	820	6.2 (5.1–7.7)	6.7 (5.4–8.3)	592	576	2.4 (2.0–2.8)	2.6 (2.1–3.2)
Male subjects								
Q1	449	96	Ref	Ref	533	220	Ref	Ref
Q2	400	176	2.1 (1.6–2.7)	2.0 (1.5–2.7)	421	227	1.3 (1.0–1.6)	1.4 (1.1–1.7)
Q3	352	324	4.3 (3.3–5.6)	4.2 (3.2–5.5)	271	189	1.7 (1.3–2.2)	1.9 (1.5–2.4)
Q4	171	328	9.0 (6.7–12.0)	8.4 (6.3–11.3)	147	288	4.7 (3.7–6.1)	5.3 (4.1–6.9)
Female subjects								
Q1	278	61	Ref	Ref	193	79	Ref	Ref
Q2	327	146	2.0 (1.5–2.9)	2.0 (1.4–2.8)	306	174	1.4 (1.0–1.9)	1.3 (0.9–1.8)
Q3	375	300	3.6 (2.7–5.0)	3.4 (2.5–4.7)	456	296	1.6 (1.2–2.1)	1.4 (1.0–1.9)
Q4	556	648	5.3 (3.9–7.2)	4.7 (3.4–6.4)	580	604	2.5 (1.9–3.4)	2.1 (1.5–2.8)
Female subjects								
Q1	275	48	Ref	Ref	189	59	Ref	Ref
Q2	319	85	1.5 (1.0–2.3)	1.6 (1.1–2.3)	302	104	1.1 (0.8–1.6)	1.1 (0.8–1.6)
Q3	363	178	2.8 (2.0–4.0)	3.1 (2.1–4.4)	439	161	1.2 (0.8– 1.7)	1.2 (0.9–1.8)
Q4	521	356	3.9 (2.8–5.5)	4.7 (3.3–6.6)	547	342	2.0 (1.5–2.8)	2.2 (1.6–3.1)

Abbreviations: 95% CI, 95% confidence interval; APC, activated protein C; ETP, endogenous thrombin potential; N, number; OR, odds ratio; Ref, reference category; TF, tissue factor; VTE, venous thromboembolism.

a ORs are relative to the reference.

b ORs are adjusted for age and sex.

## Discussion

Thrombin peak height measured at low TF was strongly associated with the first VTE (OR 6.8, 95% CI 5.5–8.3 for peak height of >75th percentile). At high TF, TG was associated with VTE but the association was less strong (OR 3.0, 95% CI 2.5–3.6 for peak height of >75th percentile). The addition of APC to slow down TG at high TF concentration, only slightly increased the association of TG measured at high TF with VTE. Calculation of the nAPCsr had no added value above peak height in the presence of APC alone. To our knowledge, this is the first study comparing three different CT-TG assay conditions within the same cohort, making direct comparisons between different assay methods possible. This study illustrated that TG performance to detect hypercoagulable states is highest when measured at low TF concentrations. The optimal TF concentration is probably achieved when the peak height in normal pool plasma is approximately 40 nM. This is likely explained by the optimal measurement of TFPI/protein S anticoagulant activity under these conditions. 12 In most studies, ETP is chosen as a main parameter of TG. Since the ETP corresponds to the area under the TG curve and reflects the total amount of generated thrombin during the entire course of coagulation it reflects the coagulation capacity of a given individual. 18 At low TF concentrations, the ETP can be overestimated by prolonged tailing of the TG curve and ETP use is not recommended. At high TF, this prolonged tailing is not observed and ETP is a reliable TG parameter. In addition, we found a stronger association with VTE for peak height over ETP, both at low and high TF concentrations (data not shown).

The association between TG and first VTE was previously examined in The Thrombophilia, Hypercoagulability and Environmental Risks in Venous Thromboembolism (THE-VTE) study, in which an elevated TG (>90th percentile) was only weakly associated with first VTE (OR 1.8 95% CI 1.2–2.7). 5 Interestingly, in the THE-VTE study, mean peak height values measured in control subjects were relatively high (∼325 nM thrombin) compared with those obtained in the MEGA study (mean peak height ∼35 nM thrombin at low TF and ∼200 nM thrombin at high TF). In the THE-VTE study, the procoagulant stimulus of TG apparently was much higher resulting in fast-onset TG and high peak heights in control subjects. This will likely have affected TG performance for detecting prothrombotic states in a negative way.

Performing TG at low TF concentrations has some drawbacks. First the lower the TF concentration used, the more crucial is the standardisation of the test. After all, the coefficient of variation of the test is higher at low TF compared with high TF. 19 In line with this, the potential influence of in vitro contract activation on the amount of thrombin generated is bigger at low TF concentrations. 20 To overcome these issues, we added TICA, a novel thermostable inhibitor of contact activation, to all plasma samples and we normalised all TG parameters against the same parameters determined in a reference plasma sample. 15

In VTE patients, it is generally accepted that routine testing for thrombophilia is not warranted as in most cases the presence of thrombophilia does not strongly predict VTE recurrence. Indeed, in the MEGA follow-up study, the VTE recurrence rate was 35% in patients with thrombophilia compared with 30% in patients without thrombophilia (OR 1.2, 95% CI 0.9–1.8). 21 As a result, clinical decision-making is not influenced by the presence of thrombophilia. Patients with unprovoked VTE have a high risk of VTE recurrence and current guidelines recommend indefinite anticoagulant therapy to most patients after first unprovoked VTE. 22 The recent American Society of Hematology guideline, however, brings about a shift in this field. The authors recommend testing for thrombophilia in patients with provoked VTE, in the context of minor risk factors (such as oral contraception). 23 In those cases, having thrombophilia could be decisive in continuing anticoagulation. In clinical practice, there might come a renewed demand for thrombophilia testing. In recent years, many attempts have been made to predict recurrent more accurately VTE risk on an individual level. Increased D-dimer level, male sex, and the presence of a residual thrombus are clinical parameters associated with an increased risk of recurrent VTE. 24 Up till now, none of these parameters are included in guidelines advising on VTE management and probably a combination of clinical parameters rather than a single test is needed to distinguish low-risk patients from high-risk patients. TG measured at low TF might help to differentiate between low- and high-risk patients. The prognostic value of TG in patients with first VTE was studied in several prospective studies. 5 25 26 27 In the AUREC study, TG was used to select patients with a low risk of VTE recurrence; patients with a low peak height (<300 nM) had lower VTE recurrence risk compared with patients with a high peak height (>400 nM [OR 0.37, 95% CI 0.21–0.66]). 25 In a study by Tripodi et al, TG in the highest tertile was associated with a 2.56 (95% CI 1.06–6.18) increased risk of VTE recurrence compared with TG in the lowest tertile. In the presence of thrombomodulin, the performance of the test was even better (OR 4.36, 95% CI 1.62–11.8). 26 Besser and coworkers did not find a difference in TG performed in the presence or absence of thrombomodulin with respect to VTE recurrence risk prediction. 27 A high ETP (>50th percentile) was associated with a 2.6-fold increased risk of VTE recurrence compared with a low ETP (<50th percentile). When thrombophilia was added, the predictive value of ETP was unchanged (HR 2.6, 95% CI 1.1–6.0) and the adjusted HR for thrombophilia was 1.1 (95% CI 0.5–2.5). Also, in the previously mentioned THE-VTE study, TG (either measured by CT or Technoclone) only had very limited value in predicting VTE recurrence. 5 In all of the abovementioned studies, peak heights were relatively high (150–350 nM). Comparing results, the best predictive value was achieved in the study of Tripodi et al when TG was measured in the presence of thrombomodulin with relatively low peak heights. We hypothesise that a further decrease in peak heights, by decreasing the procoagulant stimulus results in better predictive value of TG with respect to VTE recurrence risk. This is illustrated by a small pilot study including 74 patients with first unprovoked VTE in which an increased peak height measured at low TF was associated with a more than five-fold risk of VTE recurrence (crude OR 5.31, 95% CI 1.8–15.9). 28 Large prospective studies are needed to obtain a more accurate risk estimation.

Compared with arterial thrombosis where plaque rupture initiates thrombus formation, the initiating processes and the mechanisms by which clots are formed in the deep veins are less clear. In an autopsy study involving 50 lower extremity thrombi, no vessel wall injury was observed in 49 of them. 29 Venous thrombi are composed of fibrin-rich clots. The TF responsible for this coagulation initiation and fibrin formation is believed to originate from circulating monocytes and microparticles. 30 Thus, in contrast to arterial thrombosis in which a plaque rupture exposes high amounts of procoagulant stimuli, venous coagulation is initiated by very low amounts of TF. TFPI is the natural inhibitor of TF-induced coagulation. TFPI acts as a gatekeeper; low levels of factor Xa produced by low levels of TF/factor VIIa can be adequately eliminated by TFPI, whereas high levels of factor Xa are not. This is because TFPI inhibits TF/FVIIa and FXa by a slow tight-binding mechanism. At high TF concentrations, the generation of FXa is too fast and excessive and TFPI is overruled and loses its TG modulating capacity. However, when APC (or any other inhibitor of coagulation) is added, TG is slowed down and as a result, the TFPI/protein S anticoagulant pathway regains its ability to inhibit the slow-onset FXa and TG and to act as a TG modulator. 31 When assessing VTE risk, using slow-onset TG is more relevant as it reflects the in vivo situation.

The current study has several limitations. Blood samples from the MEGA study were kept at −80 °C for more than 15 years prior to analysis. Although case and control samples were stored and analysed under the same conditions, we cannot rule out that the ageing of the samples might have created or altered the differences in TG parameters differently between the groups. Assuring, mean thrombin peak height and ETP measured in control samples at low and high TF were comparable with those measured in normal pool plasma ( Table 1 ). Since blood samples were obtained after the occurrence of VTE, the effects of the thrombotic event on TG cannot be ruled out. Furthermore, blood samples were obtained in citrated plasma and contact activation might have occurred prior to the in vitro addition of TICA. This will, however, be the same for cases and controls. Despite several months of preparation to standardise the test and manage batch-to-batch variation, thrombin generation measured at low TF was lower (mean peak height 33 nM in normal pooled plasma) than we aimed for. This emphasises again the practical difficulties of TG with regard to its clinical utility. Nonetheless, the findings of TG experiments remain of clinical importance. Recently, a new fully automated TG analyser has been released for clinical routine laboratories. 32 It remains an open question whether this new TG method is as effective as the CT method in detecting hypercoagulable states.

In conclusion, the results of this study show that increased TG is associated with an increased risk of first VTE and that the performance of the TG test can be increased by using a low concentration of TF.

## Notes

**What is known about the Topic?**

- VTE is a multifactorial disease, in which hypercoagulability or the increased tendency to generate thrombin is believed to play an important role.
- Literature suggests an association between increased TG and first venous thrombosis.

**What Does This Paper Add?**

- TG was measured using different assay conditions simultaneously in the large cohort.
- TG, both measured at low and at high TF is associated with the first VTE; however, the association is stronger when a low concentration of TF is used.
- The sensitivity of the TG test to detect procoagulant state can be increased by using a low TF concentration.
